# Long-term risk of pneumothorax in asthmatic children: A 12-year population-based cohort study

**DOI:** 10.1097/MD.0000000000023779

**Published:** 2020-12-18

**Authors:** Chien-Heng Lin, Cheng-Li Lin, Wei-Ching Lin, Chang-Ching Wei

**Affiliations:** a Children's Hospital, China Medical University Hospital; b Department of Biomedical Imaging and Radiological Science, China Medical University; c Management Office for Health Data, China Medical University Hospital; d School of Medicine, China Medical University; e Department of Radiology, China Medical University Hospital; f School of Medicine, China Medical University, Taichung, Taiwan.

**Keywords:** asthma, children, pneumothorax

## Abstract

Pneumothorax is a life-threatening complication during acute asthma attack. However, long-term risk of pneumothorax in asthmatic children remains unknown.

In this retrospective cohort study, 333,657 children were defined as asthma cohort and a 1:1 matched non-asthma cohort were generated from 2000 to 2011. At the end of 2012, the incidence of pneumothorax in asthma and non-asthma cohorts and asthma to non-asthma hazard ratios (HRs) with confidence intervals (CIs) of pneumothorax were analyzed.

The incidence of pneumothorax was 1.35-fold higher in the asthma cohort than that in the non-asthma cohort. The asthma to non-asthma HRs of pneumothorax were higher in children younger than 6 years (1.76, 95% CI: 1.21–2.57) and in girls (2.27; 95% CI: 1.23–4.16). The HRs of pneumothorax were higher in asthmatic children with more asthma-related out-patient clinic visits/per year (>5 visits; HR: 2.81; 95% CI: 1.79–4.42), more emergency department visits/per year (>4 visits; HR: 1.68; 95% CI: 1.02–2.78), and longer hospitalization days due to asthma (>4 days; HR: 3.42; 95% CI: 1.52–6.94) (*P* < .0001, the trend test).

Asthmatic children had greater risk for pneumothorax, particularly in young children and in those with severe and uncontrolled asthma.

## 1 Introduction

Pneumothorax is a condition when air collects abnormally in the pleural space, and resulting in partial or complete collapse of the lung.^[1,2]^ Male sex, tall and thin body habitus, adolescence and young adulthood, chronic obstructive pulmonary disease (COPD), and smoking habit are well-known risk factors for pneumothorax.^[3–8]^ For decades, pneumothorax has been reported as a not common but life-threatening complication during acute asthma attack.^[9–12]^ Asthma is heterogenous, chronic airway inflammatory diseases, resulting declining airway function and tissue remodeling.^[13]^ Asthma may cause pleural bleb rupture and air leak by bronchospasm and subsequent hyperinflation.^[9–12]^ Most of previous studies about the risk factors of pneumothorax focused on the complication of acute episode of asthma.^[9–16]^ Data addressing long-term risk of asthma on the incidence of pneumothorax in children are very limited. Although reports on asthmatic patients with pneumothorax have been published for more than 30 years, the exact pathophysiology and interaction between asthma and pneumothorax remain unclear.^[9–16]^ For better understanding the influence of long-term chronic airway inflammation on the risk of pneumothorax, we performed a large population-based cohort to analyze the incidence and risks of pneumothorax in children with medical history of asthma by using data from the Taiwan National Health Insurance Research Database (NHIRD). We also investigated whether the severity and management of asthma played a role in the risk of pneumothorax.

## 2 Methods

### 2.1 Data source

The NHIRD, managed and maintained by the National Health Research Institutes, is a population-based dataset and is created from the claims data of the National Health Insurance program, a mandatory-enrolment, single-payment system generated in 1995, covering over 99% of Taiwan's population (http://www.nhi.gov.tw/english/index.aspx).^[17]^ In this retrospective, population-based study, we used the Children's file, which was derived from the NHIRD and obtained through randomly selecting 50% of all insured children in Taiwan in 2000 to 2012. Details regarding the dataset were described in our previous study.^[17–20]^ The International Classification of Diseases, 9th Revision, Clinical Modification (ICD-9-CM) was used to identify the diseases in current study.

### 2.2 Study subjects

**Figure 1:**
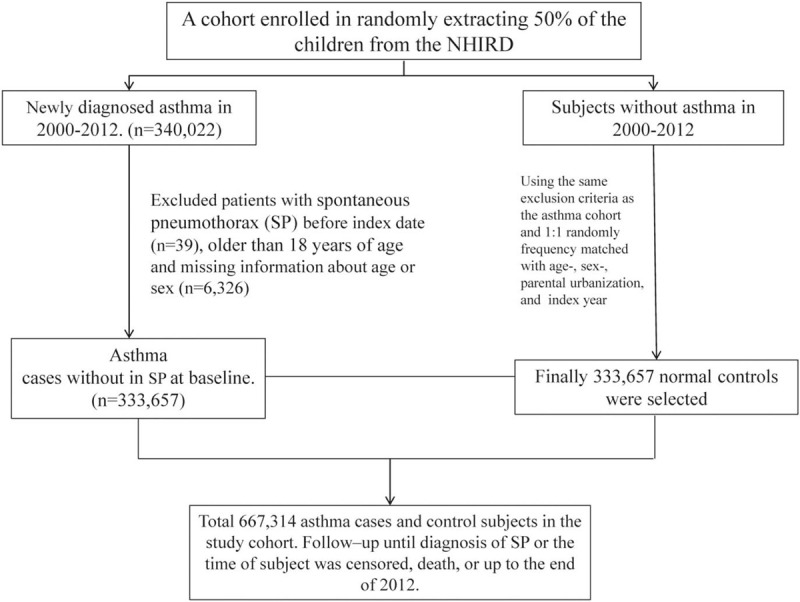
Flow diagram of the enrolment process.

In this study, we aimed to analyze the incidence rate (IR) and relative risk (incidence rate ratio) of pneumothorax in asthma and non-asthma cohorts in 2000 to 2012. Asthma was defined as children younger than 18 years with at least 3 ambulatory claims or 1 inpatient claim in any diagnostic field using the ICD-9-CM code 493. A total of 333,657 children newly diagnosed with asthma between 2000 and 2011 were identified as the asthma cohort. The diagnosis of asthma was made by allergists, pulmonologists, and family doctors who have completed Taiwan Asthma Certification Course. To improve diagnostic accuracy and avoid overestimation of incidence, children with asthma were defined as at least 3 records with respective ICD-9-CM codes in any diagnosis field of inpatient claims or ambulatory claims and those treated with regular controller medications, including inhaled corticosteroid (ICS), Montelukast, or both, were defined those with continuous medication for at least 3 months. The validation of asthma diagnosis in current study corresponding to previous studies, such as the International Study of Asthma and Allergies in Childhood (ISAAC), has been discussed in our previous paper.^[18]^ The baseline index date was the first date of asthma diagnosis. For children without asthma, we randomly selected 1 non-asthmatic child, who never had ICD-9-CM code 493 in any diagnostic field, matched by age, sex, urbanization level, and baseline year. Children with missing data or those with preexisting pneumothorax (ICD-9-CM code 512.0, 512.1, 512.8) before the baseline year were excluded. Some diseases can be confused with asthma, such as cystic fibrosis, congestive heart failure, primary ciliary dyskinesia, emphysema, immunodeficiency conditions vocal cord dysfunction, or airway and vascular malformations, were excluded. We also excluded patients who were simultaneously diagnosed (coded) with asthma and pneumothorax in the ambulatory or inpatient claim. Each child was followed up from the index date until pneumothorax development, insurance withdrawal, or conclusion of follow-up on December 31, 2012. The flowchart in Figure 1 summarized the enrolment process.

### 2.3 Statistical analysis

The sociodemographic characteristics in this study included age, sex, and urbanization level. The degree of urbanization of a population was categorized into 4 levels: level 1, the densest and level 4, the least dense.^[19]^ All analyses were performed using the with SAS software (version 9.1, SAS institute Inc., Carey, NC). The level of significance was set at *P* < .05 in two-tailed tests. The means and standard deviations (SDs) for continuous variables and counts and percentages for categorical variables were used to show the distributions of asthma and non-asthma cohorts for the baseline. Differences were analyzed using the chi-square test for categorical variables and Student's test for continuous variables. The Kaplan–Meier estimate was used to estimate the proportion of study subjects who did not suffer from pneumothorax of both cohorts during the follow-up period. The incidence rate of pneumothorax is shown as the number of newly diagnosed pneumothorax per person-years in both cohorts. Hazard ratios (HRs) and 95% confidence intervals (CI) were calculated using multivariate Cox proportional hazard regression models, with the non-asthma control cohort as the reference group, to assess the association between asthma and the risk of pneumothorax. We also used the Cox proportional hazards model to estimate the HRs of pneumothorax based on the medications for asthma, the frequency of asthma-related medical visits, emergency department (ED) visits, and length of hospitalization.

## 3 Results

**Figure 2:**
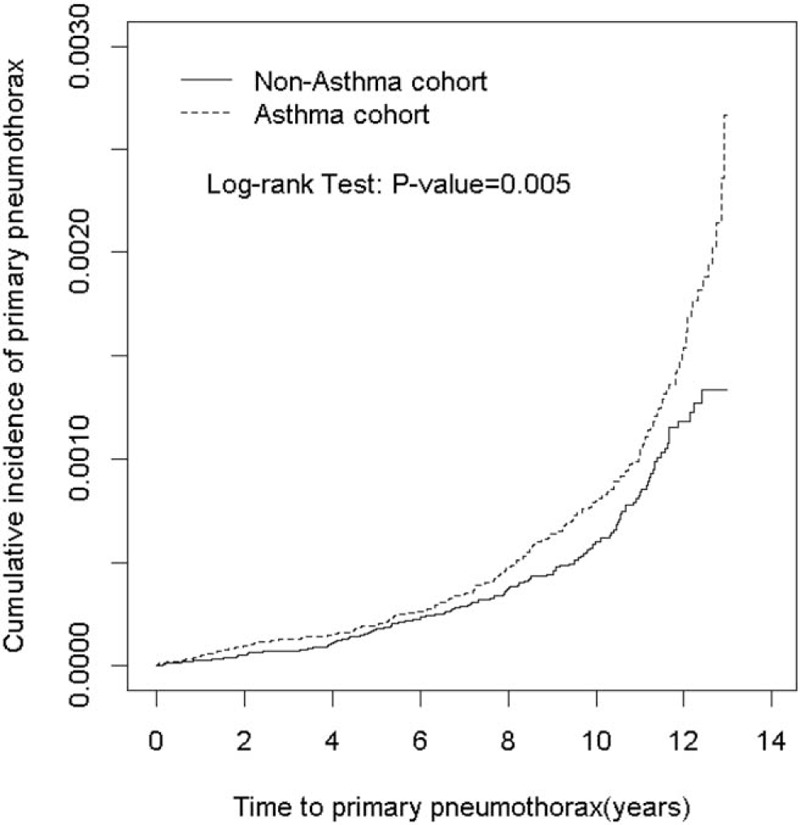
The Kaplan–Meier analysis of cumulative incidence of pneumothorax for asthma cohort compared to non-asthma cohort.

This study evaluated 333,657 asthma cases and 333,657 non-asthma controls. No differences in the sociodemographic characteristics were detected between the 2 study groups. Majority of children with asthma were boys (58.1%), aged <6 years (79.0%), and lived in urbanized areas (62.2%) (Table 1). The mean follow-up periods were 7.7 (SD = 3.16) years and 7.69 (SD = 3.16) years for the asthma and non-asthma cohorts, respectively. The mean years from asthma to pneumothorax diagnoses were 6.99 (SD = 3.64) for the asthma cohorts. Kaplan–Meier analysis demonstrated that the accumulated incidence rate of pneumothorax was significantly higher in the asthma cohort than non-asthma cohort (log-rank test *P* < .0001; Fig. 2).

**Table 1:** Demographics between children with and without asthma.

	Non-asthma (N = 333,657)	Asthma (N = 333,657)
	n (%)	n (%)
Age, yr, mean (SD)	4.38 (3.06)	4.38 (3.04)
Stratified age, yr		
<6	263,533 (79.0)	263,533 (79.0)
6–11	60,113 (18.0)	59,886 (18.0)
≥12	10,011 (3.00)	10,238 (3.07)
Sex		
Girl	139,669 (41.9)	139,669 (41.9)
Boy	193,988 (58.1)	193,988 (58.1)
Urbanization level^∗^		
1 (highest)	102,666 (30.8)	102,666 (30.8)
2	104,759 (31.4)	104,759 (31.4)
3	61,092 (18.3)	61,092 (18.3)
4 (lowest)	65,140 (19.5)	65,140 (19.5)
Mean follow-up period, yr, mean (SD)	7.69 (3.16)	7.70 (3.16)
Mean time from asthma to pneumothorax, yr, mean (SD)	6.87 (3.38)	6.99 (3.64)

The incidence densities of pneumothorax for both cohorts and stratified with sociodemographic status are presented in Table 2. At the end of a 12-year follow-up, pneumothorax incidence was found to be 1.35-fold higher (95% CI: 1.10–1.66) in the asthma cohort than that in the non-asthma cohort (8.25 vs 6.12 per 100,000 person-years). Comparisons between genders showed that pneumothorax incidences were higher in boys in both cohorts. Girls had higher adjusted HRs (2.27, 95% CI: 1.23–4.16) of pneumothorax than boys had (1.26, 95% CI: 1.01–1.57). Comparisons between age stratification showed that pneumothorax incidences were higher in older children in both cohorts. However, children younger than 6 years had highest adjusted HRs (176, 95% CI: 1.21–2.57) of pneumothorax than those older than 6 years. Compared to the non-asthma cohort, the adjusted HR increased with the number of annual asthma-related out-patient clinic visits, from 1.19 (95% CI: 0.96–1.48) for those with ≤3 visits to 2.81 (95% CI: 1.79–4.42) for those with >5 visits (*P* < .001 for trend) (Table 3). Compared to the non-asthma cohort, the adjusted HR increased with the number of annual ED visits due to asthma, from 1.31 (95% CI: 1.06–1.62) for those with ≤2 visits to 1.68 (95% CI: 1.02–2.78) for those with >4 visits (*P* < .001 for trend). In addition, the asthma cohort with more frequent admissions due to asthma (>4 times/year) had higher adjusted HR (3.24; 95% CI: 1.52–6.94) of pneumothorax compared to non-asthma controls (*P* for trend <.001) (Table 3). Asthmatic children treated only with leukotriene receptor antagonist (LTRA) had higher adjusted HR (3.24; 95% CI: 1.52–6.94) of pneumothorax than those treated with only ICS or with combined ICS and LTRA (Table 4).

**Table 2:** The risk of pneumothorax in children with asthma compared to children without asthma stratified by age and gender in Cox proportional hazard regression.

	Non-asthma			Asthma			
	Event	Person-years	IR	Event	Person-years	IR	Adjusted HR^†^ (95% CI)
All	157	2,564,179	6.12	212	2,570,020	8.25	1.35 (1.10, 1.66)^∗∗^
Age, yr							
<6	42	2,136,783	1.97	75	2,142,227	3.50	1.76 (1.21, 2.57)^∗∗^
6–11	81	396,216	20.4	102	395,380	25.8	1.27 (0.95, 1.69)
≥12	34	31,180	109.0	35	32,413	108.0	0.98 (0.61, 1.57)
Sex							
Girl	15	1,060,303	1.41	34	1,062,688	3.20	2.27 (1.23, 4.16)^∗∗^
Boy	142	1,503,875	9.44	178	1,507,332	11.8	1.26 (1.01, 1.57)^∗^

**Table 3:** The risk of pneumothorax among average frequency of medical visits for asthma in Cox proportional hazard regression.

Average frequency of medical visit, per year	Event	Person-years	IR	Adjusted HR^†^ (95% CI)
Frequency of out-patient clinic visits/per year				
Non-asthma	157	2,564,179	6.12	1.00 (Reference)
≤3	164	2,162,768	7.58	1.19 (0.96, 1.48)
4–5	25	228,398	11.0	2.30 (1.51, 3.52)^∗∗^
>5	23	178,853	12.9	2.81 (1.79, 4.42)^∗∗^
*P* for trend				<.001
Frequency of ED visits/per year				
Non-asthma	157	2,564,179	6.1	1.00 (Reference)
≤2	180	2,230,557	8.1	1.31 (1.06, 1.62)^∗^
3-4	15	165,286	9.1	1.66 (0.98, 2.83)
>4	17	174,177	9.8	1.68 (1.02, 2.78)^∗^
*P* for trend <.001				<.001
Frequency of hospitalizations/per year				
Non-asthma	157	2,564,179	6.1	1.00 (Reference)
≤2	199	2,346,586	8.5	1.32 (1.07, 1.63)^∗∗^
3–4	6	138,672	4.3	1.52 (0.67, 3.44)
>4	7	84,762	8.3	3.24 (1.52, 6.94)^∗∗^
*P* for trend <.001				<.001

**Table 4:** The risk of primary pneumothorax among asthma children with different control medications compared to non-asthma controls in Cox proportional hazard regression.

Control medications for asthma	N	Event	IR	Adjusted HR^†^ (95% CI)
Non-asthma	333,657	157	0.61	1.00
Asthma treated with				
1. ICS	96,209	72	0.88	1.25 (0.94, 1.65)
2. LTRA	49,680	16	0.51	1.71 (1.02, 2.86)^∗^
3. ICS + LTRA	75,840	25	0.42	1.26 (0.83, 1.93)

## 4 Discussion

The incidence of spontaneous pneumothorax is between 6 and 18 per 100,000 in the general population.^[1,3]^ The exact incidence of primary in children is unknown, but pneumothorax has been reported throughout the childhood period.^[5,14,16]^ This is one of the few population-based cohort studies that demonstrate an association between childhood asthma and the risk of pneumothorax. Based on the 12-year observation, children with asthma had 35% increased risks of pneumothorax, regardless age, sex, and urbanization level. Girls with asthma and early childhood asthma had greater risks of pneumothorax. Moreover, children with more asthma-related medical visits, ED visits, and admissions were highly at risk for pneumothorax.

Rapid urbanization and advent of high-speed motor vehicles results in environmental degradation and air pollution. Air pollution had a significant impact on health-care expenditure on respiratory diseases.^[21]^ Air pollution is a well-known cause which can worsen asthma symptoms.^[21]^ Increased concentration of air pollutants has also been reported significantly associated with increased risks of pneumothorax. Hence, level of urbanization is a potential confounder in current study.

Spontaneous pneumothorax is divided to primary and secondary.^[1,6]^ The cause of primary spontaneous pneumothorax is not clear, but some risk factors, including male sex, smoking, and a family history of spontaneous pneumothorax, have been reported.^[3,5,6]^ Secondary pneumothorax often occurs in patients with parenchymal pulmonary disease, including asthma, COPD, infections of the lung, interstitial lung disease, sarcoidosis, idiopathic pulmonary fibrosis, pulmonary Langerhans’ cell histiocytosis, lymphangioleiomyomatosis, lung cancer, and certain congenital malformations.^[1,5–7]^ Several systemic diseases are also infrequently associated with pneumothorax, such as anti-neutrophil cytoplasmic antibody (ANCA)-associated vasculitis, systemic lupus erythematosus (SLE), rheumatoid arthritis, scleroderma, and dermatomyositis.^[1,5]^ Asthma is a common chronic airway disease, but very few studies assessed the incidence and risk of spontaneous pneumothorax of asthmatic children on a large-scale basis.

Despite advances in asthma treatment, asthma remains the most common chronic diseases in children and a leading cause of morbidity. Pneumothorax is a well-known and life-threatening complication during asthma with acute exacerbation.^[9–12]^ During asthma with flare, bronchospasm and hyperinflation increase the pleural pressures, which may lead to pleural bleb rupture and pneumothorax.^[8]^ To date, the long-term effect asthma on the incidence of pneumothorax in children remains unclear. Only 2 case studies have reported that pneumothorax is not an uncommon complication of asthma and that its incidence of pneumothorax corresponds to that of the general population.^[14,16]^ Our finding is inconsistent with those of the previous studies. We found that children with asthma had even higher incidence (1.35-fold higher) and risk of pneumothorax than non-asthma controls. The increased risk of pneumothorax was particularly seen in asthmatic children younger than 6 years, in girls, and in those with severe and uncontrolled asthma.

From literature, 11.5% of patient with spontaneous pneumothorax have a family history of pneumothorax. The hereditary conditions, including Marfan syndrome, homocystinuria, Ehlers–Danlos syndromes, alpha 1-antitrypsin deficiency, and Birt–Hogg–Dubé syndrome, have all been linked to familial pneumothorax.^[1,5]^ However, family history and genetic information are not available in our dataset.

Pneumothorax is believed to result from the spontaneous rupture of a subpleural bleb or bulla.^[22]^ However, only a few patients with pneumothorax had blebs or bullae in computed tomography imaging or at the time of surgery.^[22]^ Alternative mechanisms may be related to increased pleural porosity secondary to inflammation.^[23]^ The development of blebs, bullae, or pleural porosity might be associated with many factors, such as distal airway inflammation, distal bronchial tree anomaly, connective tissue formation disorders, local ischemia, and malnutrition.^[2]^ Asthma is a complex and chronic inflammatory disorder associated with airway hyperresponsiveness and tissue remodeling of the airway structure.^[24]^ Asthma with exacerbation is a predisposing factor for pneumothorax development, which could be a potentially fatal complication and may be missed due to its rarity and non-obvious diagnosis.^[8,25]^ In this study, the risk of pneumothorax increased in children with asthma who had more outpatient and ED visits and longer hospitalization days. It implies uncontrolled and severe airway inflammation related to increased risk of pneumothorax.

In adult studies, spontaneous pneumothorax has been reported more frequent in men than in women and female to male ratio is ranging from 1:2 to 1:6.^[1,5]^ However, incidence and distribution of pneumothorax with respect to age and sex in children remain elucidative. In our study, male patients with or without asthma had subsequently more pneumothorax events; however, female patients with asthma had a higher risk for pneumothorax than those without asthma, especially those aged <6 years. The reasons for sex-related differences are not known in children. The mechanism of age-related differences may be caused by chronic airway inflammation, bronchospasm, and hyperinflation that increase the pleural pressures needed to maintain ventilation, which could induce potential pleural bleb rupture in children with asthma, resulting in pneumothorax. The longer the duration of airway inflammation, which may occur in patients aged <6 years, the higher the risk of subsequent pneumothorax.

In this study, asthmatic children treated only with montelukast had higher risk of pneumothorax than those treated with ICS or with combined ICS and montelukast. All current international guidelines recommend the use of low-dose ICS as the preferred controller therapy, with LTRA as an alternative, for the management of persistent asthma in children (5–11 years of age) and adolescents.^[26–28]^ ICS shows a better anti-inflammatory effect and most children and adolescents with mild to moderate persistent asthma treated with ICS had better lung function and asthma control than with LTRA.^[26–28]^ This may explain why asthmatic children treated with LTRA had higher risk of pneumothorax than those treated with ICS.

The strength of this study is the precise analysis of the future risk of pneumothorax in children with asthma based on a large population database with minimal selection bias. However, this study has several limitations. First, asthma and pneumothorax were diagnosed based on ICD-9-CM codes; data on disease severity, parental smoking, family history of atopy and pneumothorax, genetic information, chest radiographs, and immunoglobulin E (IgE) or specific IgE levels were not available. Second, problems related to coding accuracy and financial incentives may also lead to bias when using ICD-9 codes for diagnosis in large insurance claims data for research. These potential limitations are partly countered by the strengths of the large sample size of children with asthma in this study. Third, the period of study was only 13 years. However, this is the longest observation study with a large population to date.

## 5 Conclusion

In this retrospective population-based cohort study, we found that childhood asthma might be a risk factor for pneumothorax, especially in girls, asthmatic children younger than 6 years, and those treated with montelukast and those having more asthma-related medical visits. Further well-designed studies are required to evaluate the effect and pathophysiology of asthma on the development of pneumothorax.

## Author contributions

CH Lin, CC Wei designed the study and prepared the manuscript, CL Lin and WC Lin collected the data, CL Lin made the data analysis. All authors read and approved the final manuscript.

**Conceptualization:** Chien-Heng Lin, Wei-Ching Lin, Chang-Ching Wei.

**Data curation:** Wei-Ching Lin.

**Formal analysis:** Cheng-Li Lin, Wei-Ching Lin.

**Funding acquisition:** Chien-Heng Lin.

**Investigation:** Chien-Heng Lin, Chang-Ching Wei.

**Methodology:** Cheng-Li Lin, Chang-Ching Wei.

**Resources:** Wei-Ching Lin.

**Software:** Cheng-Li Lin.

**Validation:** Wei-Ching Lin.

**Visualization:** Wei-Ching Lin.

**Writing – original draft:** Chang-Ching Wei.

**Writing – review & editing:** Chang-Ching Wei.
